# Effect of eye shield and ear muffs on pain intensity during venous blood sampling in premature infants: a clinical trial study

**DOI:** 10.1186/s12887-023-03978-3

**Published:** 2023-04-06

**Authors:** Fatemeh Shykhveisi, Roghayeh Jafarian Amiri, Ali Zabihi, Mohsen Haghshenas Mojaveri, Afsaneh Arzani, Mohammad Chehrazi, Zahra Valizadeh Chari

**Affiliations:** 1 Fatemeh Shykhveisi (MSc), Student Research Committee, Babol University of Medical Sciences, Babol, Iran; 2 Department of Medical & Surgical Nursing, School of Nursing & Midwifery, Babol University of Medical Sciences, Babol, Iran; 3 Social Determinants of Health Research Center, Health Research Institute, Babol University of Medical Sciences, Babol, Iran; 4 Department of Pediatrics, School of Medicine, Non-Communicable Pediatric Disease Research Center Health Research Institute Amirkola Hospital Babol University of Medical Sciences, Babol, Iran; 5 Non-Communicable Pediatric Diseases Research Center, Health Research Institute, Babol University of Medical Sciences, Babol, Iran; 6 Department of Epidemiology and Biostatistics, School of Health, Babol University of Medical Sciences, Babol, Iran; 7 Clinical Research Development Unit of Rouhani Hospital, Zahra Valizadeh Chari (BS), Babol University of Medical Sciences, Babol, Iran

**Keywords:** Blood sampling, Eye shield, Ear muffs, Pain management, Premature infants, Clinical trial

## Abstract

**Background:**

Today, due to the side effects of drugs, there is a greater desire to use non-pharmacological interventions to relieve pain caused by painful procedures. Using non-pharmacological interventions in combination is more effective than using them alone in relieving the pain of infants. Reducing sensory and environmental stimuli such as visual and auditory stimuli is one of the non-pharmacological methods to relieve pain. The aim of this study was to investigate the effect of using eye shield and ear muffs on pain intensity during venous blood sampling of premature infants.

**Methods:**

In this clinical trial study, 148 premature neonates admitted to the Neonatal Intensive Care Unit of Rouhani and Children Hospitals in Babol were randomly assigned to four groups of 37. Fifteen minutes before intravenous blood sampling until 15 min later, in the first group, eye shield; in the second group, ear muffs, and in the third group, eye shield plus ear muffs were used. In the fourth group (control), blood sampling was performed routinely. NIPS pain scale and demographic questionnaire were used to collect the data.

**Results:**

The results showed that during the venous blood sampling was a significant difference between the mean pain intensity of neonatal in the eye shield plus ear muffs group (3.14 ± 0.71), the ear muffs group (4.43 ± 1.21), the eye shield group (5.41 ± 1.04).) and the control group (5.94 ± 0.84) (P = 0.001). Moreover, after the venous blood sampling, there was a significant difference between the mean neonatal pain intensity in the eye shield plus ear muffs group (1.19 ± 0.39), the ear muffs group (1.43 ± 0.50), the eye shield group (1.33 ± 0.37) and the control group (1.89 ± 0.90) (P = 0.001).

**Conclusions:**

In this study, the pain severity during and after venous blood sampling in the ear muffs plus eye shield was lower than in other groups. Therefore, a combination of ear muffs and eye shield is recommended as a better pain reliever when performing venous blood sampling in premature infants.

## Introduction

Newborns’ ability for pain modulation is low, and they are unable to secrete dopamine and norepinephrine to modulate pain until 36–40 weeks of pregnancy [1]. Early and continuous exposure to painful stimuli before 38 to 40 weeks in premature infants leads to permanent behavioral changes, increased intracerebral pressure, immunosuppression, and cardiac arrhythmia [2]. Furthermore, pain caused by painful procedures in very low birth weight infants (VLBW) may lead to brain development disorders. Therefore, it is necessary to reduce the pain of premature babies in order to support physiological stability and prevent long-term effects related to painful experiences [3].

The term pain is defined as an unpleasant emotional sensation or experience in connection with potential or actual tissue damage in the body [4]. Pain in infants is considered a deadly feeling, and all health caregivers should be aware of the category of pain and make efforts to prevent or control it in infants (especially premature babies) [5].

Painful procedures can adversely affect the infant’s physiological indicators, such as plasma cortisol level, blood oxygen saturation, heart and breath rates, and behavioral parameters, such as crying, smiling, and body movements [6]. In addition, a painful intervention in the NICU can have long-term effects on the infant’s neurological and behavioral development, such as anxiety, stress, and distraction during adolescence and adulthood [7].

Venous blood sampling is one of the most common painful invasive procedures when caring for NICU infants [8]. Pain is often overlooked in the NICU because infants are unable to complain of pain. Since premature infants are at risk of infection, intensive care unit nurses are forced to change the location of the venous catheter every 3–4 days to prevent infection [9].

So far, two pharmacological and non-pharmacological methods have been used to control pain in premature infants. Non-pharmacological methods include non-nutritive sucking, breastfeeding, oral solutions such as sucrose and glucose, and skin-to-skin contact between the infant and the mother [5, 10]. More recent studies have concluded that controlling sound and light as two physical stimuli affects the changes in infants’ electroencephalogram. Therefore, by reducing disturbing physical stimuli, pain responses in these infants can be reduced [11]. These studies showed that the light of the NICU environment could affect infants’ responses to pain during painful procedures, which could be reduced by using an eye shield [12, 13].

The evidence indicates a decrease in the pain response due to a decrease in environmental sensory stimulation in premature infants. Consequently, studies have shown that reducing infants’ exposure to light and sound in the NICU environment by using eye shield and ear muffs can strengthen infants’ physiological stability [14]. On the other hand, it has been stated in a study that the fetus is able to recognize the sound and has the power of sound patterning in the mother’s womb. Due to this, newborn infants have a greater preference for hearing their mother’s voice after birth. The infant’s response to the mother’s voice is associated with heart rate changes, and after about two minutes of listening to the mother’s voice, the infant’s heart rate decreases significantly [15].

Mater et al.‘s study showed that using eye shield and massage was associated with a significant decrease in pain response compared to the control group during blood sampling [16]. A study by Aita et al. showed that infants with eye shield and ear muffs before the heel blood collection did not significantly differ in pain perception during the procedure compared to control group infants [17]. Therefore, only in some of these studies, the effect of using these interventions on reducing the pain of babies has been confirmed and more studies are needed to prove these findings.

In recent years, concern about the side effects of drugs has led to a greater desire to use non-pharmacological interventions to relieve pain caused by painful procedures [15]. On the other hand, studies have shown that the use of non-pharmacological interventions for pain relief in infants in combination with other non-pharmacological interventions is more effective than the use of non-pharmacological interventions alone in pain relief [18, 19]. Since in most previous studies, non-pharmacological interventions, especially the use of eye shield and ear muffs alone, have been used to relieve the pain of infants hospitalized in the NICU, considering their simplicity and safety, this study aims to investigate the effect of eye shield and ear muffs was performed alone and in combination to reduce pain caused by venous blood sampling in hospitalized infants.

In general, the findings of the studies are contradictory [16] and further studies are needed to prove these findings. In addition, using non-drug methods as a simple and safe method can be useful in reducing the pain of infants. Therefore, this study was conducted to investigate the effect of using eye shield and ear muffs in premature infants in reducing pain intensity during venous blood sampling.

## Materials and methods

The present clinical trial study was conducted in 2021 on 148 premature Infants admitted to the Neonatal Intensive Care Units (NICU) of Rouhani and Children’s Hospitals in Babol with the permission of the Ethics Committee of Babol University of Medical Sciences (code IR.MUBABOL.HRI.REC.1398.188) and informed consent. Sampling in the first stage was done as convenience. Then, the infants were assigned to four groups of 37: control, eye shield, ear muffs, and eye shield plus ear muffs based on the random allocation method. With applying the inclusion and exclusion criteria, infants were assigned into groups using the permuted block randomization method. The randomization unit included infants, and the block size was 4. To conceal random allocation, a list generated by a statistician was used. The assigned group of each subject had a specific code that was a combination of numbers and letters with no special order. This list was not provided to the principal of the study (researcher) and the person responsible for the allocation [21].

In this study, inclusion criteria include: premature infants with a gestational age of 28 to 37 weeks, Apgar above 7 in 5 min, no painful procedure at least 6 h before blood sampling, Sampling should be done when the baby is awake and active and in between feedings. Also, the exclusion criteria include premature infants with congenital problems, not using narcotic and antidepressants for the mother during pregnancy and not using analgesic in infants, 12 h before blood sampling. This study was registered in Iran’s clinical trial site with number IRCT20200913048704N1.

**Fig. 1:**
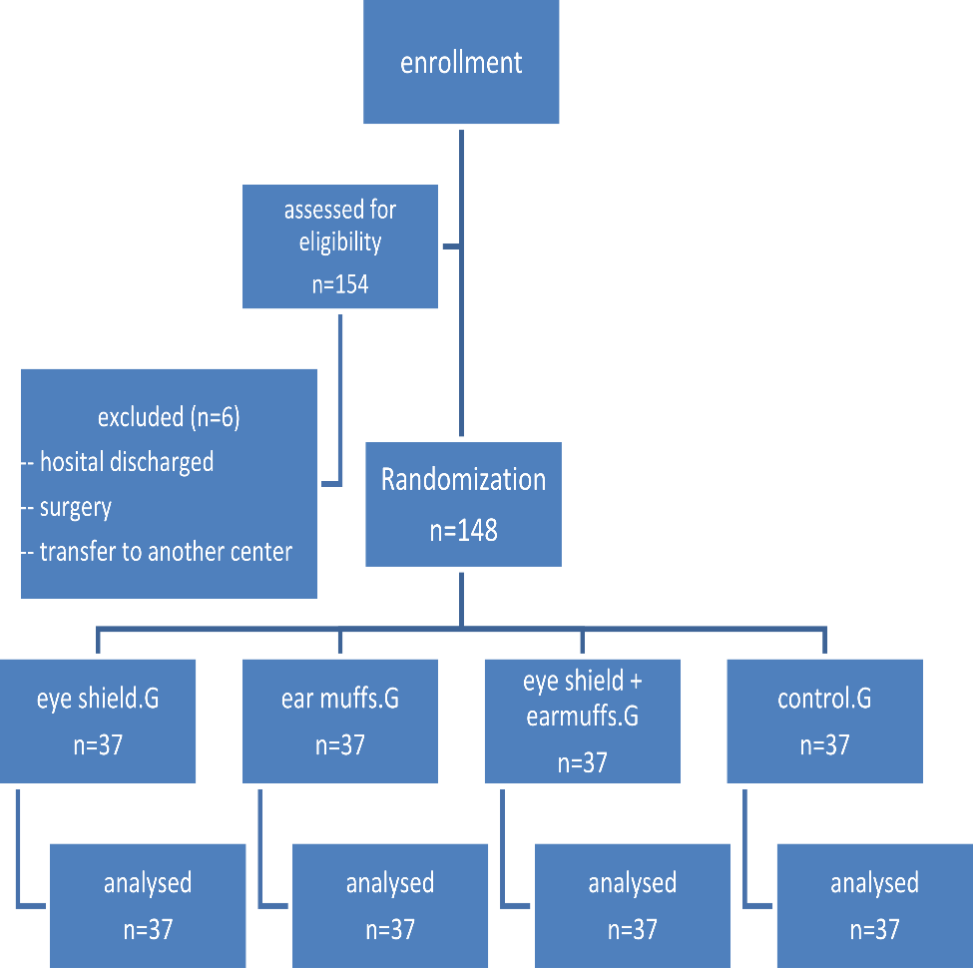
Selection of the study participants

The sample size were based on the previous study [22] and using the formula, taking into account the average NIPS score of premature infants after blood sampling and using the NCSS version 11 software. Considering the alpha coefficient of 0.0125 and beta of 20%, the required sample size of 37 premature infants in each group and a total of 148 eligible samples were randomly selected in 4 equal groups (37 in each group). Sampling was done in a period of eight months (Fig. 1). The primary outcome was pain intensity during blood sampling and the secondary outcome was physiological changes in infants during and after blood sampling.

Infants’ venous blood sampling were performed following the NICU doctors’ order according to the clinical goals. In each study group, using an eye shield and ear muffs started 15 min before blood collection and continued until 15 min after blood collection in order to investigate its effect on the intensity of blood collection pain [8]. In the control group, neither the ear muffs nor eye shield was used. In this study setting, infants routinely receive no (non) pharmacological intervention during venous blood sampling. The intervention was implemented when the infant was awake, and the eye shield only covered the infant’s eyes. The way of covering the incubator and its environment was similar in all four groups regarding sound and light. Eye shields used in the intervention group were made in Iran by the company of Tajhiz Yaran (Ortoteb) and the ear muffs, model Elvex, were made in the United States ( Reduces noise by at least 25%).

The data collection tool included demographic profile questionnaire and NIPS pain measurement scale. Demographic characteristics included: gestational age, gender, weight, height, infant’s head circumference, hospitalization date, disease diagnosis, and mother’s type of delivery. The researcher completed the first part of the questionnaire before sampling. In this study, pain response was recorded by the researcher’s assistant based on the NIPS scale and the infant’s behavioral and physiological changes (respiratory pattern) by videotaping the infant 15 min before, during, and 15 min after blood sampling. During the video recording, the camera was focused on the infant. In addition, the interpretation of the video was performed by the researcher and her assistant. Blood sampling was performed by a nursing expert in each hospital who had at least one year of experience in the NICU, with a single entry of the needle gauge24-26. In order to simultaneously observe the infant’s face, the video recording was performed by the researcher’s assistant. Heart rate (HR) and its changes (VHR) (measured within 30 s) 15 min before, during, and 15 min after blood sampling, as well as oxygen saturation, were checked using a cardiorespiratory monitoring device.

This study evaluated pain in infants using the Neonatal Infant Pain Scale (NIPS). This tool has six items, 5 of which are behavioral (facial movements, crying, movements of hands, legs, and state of consciousness), and one is physiological (breathing pattern). The total score obtained in this tool is between 0 and 7. This tool is used for both groups of full-term and premature infants and is generally used to evaluate pain caused by heel bleeding and venous or arterial puncture and pain after surgery [23].

In this scale, six items are examined:

1- Facial expression (zero for a relaxed state and one for a tense and frowning state). 2- Infant crying (zero for no crying, 1 for whining, and 2 for intense crying). 3- Breathing pattern (zero for relaxed state and one for breathing change). 4- Movement of the hands (zero for relaxed state and one for folding or opening). 5- Movement of the legs (zero for relaxed or lying down and one for folding or unfolding). 6- State of consciousness (zero for sleep or wakefulness and one for screaming and shouting). In general, a score of 0–3 indicates no pain, a score of 4–5 indicates moderate pain, and a score of 6–7 indicates severe pain [23]. The validity and reliability of this tool have been investigated and confirmed in the studies by Daily et al. [24] and Bromandfar et al. [23] (r = 0.93).

After collecting the data, it was entered into SPSS software version 23. Chi-Square was used to measure qualitative variables and t-test was used to measure quantitative variables. Using repeated measurement test and analysis of variance (ANOVA), the difference in pain score, HR and VHR and behavioral changes at different times were investigated.

## Results

In this study, 148 premature infants were studied, of whom 71 (47.97%) were girls and 77 (52.03%) were boys. Mean gestational age and weight were 34.15 ± 1.52 weeks and 2124.47 ± 582.48 g, respectively. Ninety-nine deliveries (66.89%) were cesarean section, and 49(33.11%) were vaginal. Infants did not significantly differ in terms of sex, weight, height, head circumference, type of delivery, and gestational age (*p* > 0.0 5).

The results showed that during and after blood sampling, heart rate changes were significant among the studied groups (p = 0.001). Pairwise comparisons of the groups showed that infants’ heart rates during and after blood sampling was significantly different between eye shield and the control group, ear muffs and the control group, eye shield plus ear muffs and the eye shield group, and ear muffs and the control group (p = 0.001) (Table 1).

**Table 1:** Comparison of infants’ heart rate of study groups in before, during, after blood sampling

Stages Groups	Before Mean ± SD	during Mean ± SD	after Mean ± SD
Eye shield (n = 37)	135.49 ± 12.42	169.27 ± 19.91^a^	137.14 ± 16.94^a^
Ear muffs (n = 37)	136.86 ± 12.05	164.48 ± 15.71^a^	140.22 ± 15.45^a^
(n = 37)Eye shield plus ear muffs	133.38 ± 8.66	157.73 ± 9.46^a,b,c^	152.65 ± 9.28^a,b,c^
Control (n = 37)	139.05 ± 10.75	167.35 ± 6.21	161.70 ± 5.50
P value	0.330	0.001	0.001

a: significantly different compared to the control group, b: significantly different compared to the eye shield group, c: significantly different compared to the ear muffs group

The results showed a significant difference in the percentage of oxygen saturation among the infants of the studied groups during blood sampling (p = 0.008) and after blood sampling (p = 0.003). Besides, pairwise comparisons of the groups showed that the percentage of saturated oxygen during blood sampling in the ear muffs group and the ear muffs plus eye shield was significantly different from the control group (p = 0.001). Moreover, the percentage of saturated oxygen after blood sampling in each of the eye shield, ear muffs, and eye shield plus ear muffs groups was significantly different from the control group (p = 0.003) (Table 2).

**Table 2:** Comparison of infants’ oxygen saturation percentage of study groups in before, during, after blood sampling

Stages Groups	before Mean ± SD	during Mean ± SD	after Mean ± SD
Eye shield (n = 37)	96.7 ± 1.96	94.35 ± 6.37	97.16 ± 2.02^a^
Ear muffs (n = 37)	96.65 ± 1.27	96.30 ± 2.83^a^	97.43 ± 2.21^a^
(n = 37)Eye shield ear muffs	96.51 ± 1.93	96.37 ± 2.34^a^	95.73 ± 1.44^a^
Control (n = 37)	96.3 ± 1.17	95.78 ± 2.05	93.73 ± 1.92
P value	0.400	0.008	0.003

a: significantly different compared to the control group, b: significantly different compared to the eye shield group, c: significantly different compared to the ear muffs group

The results showed significant changes in the infants’ pain among the studied groups during and after blood sampling (p = 0.001). Pairwise comparisons of the groups showed that during the blood sampling, the infants’ pain level in the ear muffs group was significantly different from the control group and eye shield group (p = 0.001). Moreover, the pain level in the eye shield plus ear muffs group significantly differed from the control, eye shield, and ear muffs groups (p = 0.001). In addition, after blood sampling, the comparison of the pain levels of the infants in the eye shield, ear muffs, and ear muffs plus an eye shield groups showed a significant difference with the control group (p = 0.001) (Table 3).

**Table 3:** Comparison the mean infants’ pain of study groups in before, during, and after blood sampling

Stages Groups	Before Mean ± SD	during Mean ± SD	after Mean ± SD
Eye shield (n = 37)	1.08 ± 0.36	5.41 ± 1.04	1.16 ± 0.37^a^
Ear muffs (n = 37)	1.32 ± 0.70	4.43 ± 1.43^a,b^	1.43 ± 0.50^a^
Eye shield ear muffs (n = 37)	1.49 ± 0.55	3.14 ± 0.71^a,b,c^	1.19 ± 0.39^a^
Control (n = 37)	1.30 ± 0.87	5.94 ± 0.84	1.89 ± 0.90
P value	0.070	0.001	0.001

a: significantly different compared to the control group, b: significantly different compared to the eye shield group, c: significantly different compared to the ear muffs group

**Fig. 2:**
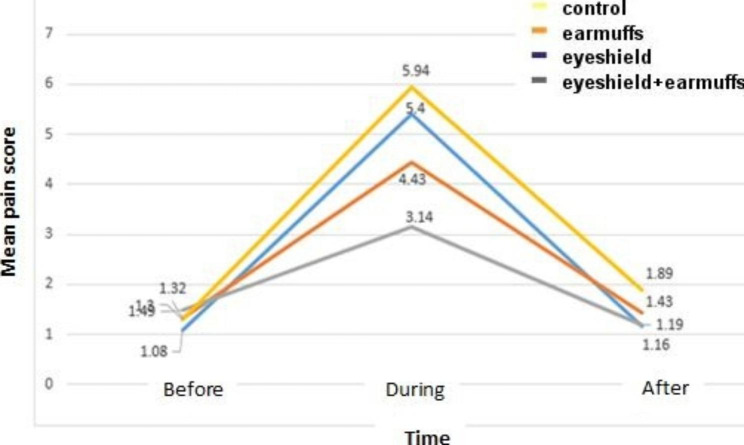
Comparison of infant pain changes before, during, and after blood sampling in study groups

According to Table 3; Fig. 2, the trend of pain changes in the eye shield plus ear muffs group was lower than in other groups.

## Discussion

This study showed that during and after blood sampling, the changes in the infants’ pain were significant among the study groups. So that during the blood sampling, the pain intensity of the infants in the ear muffs group with the eye shield group, the ear muffs group with the control group, and the eye shield plus ear muffs group were significantly different from each of the other groups. After blood sampling, there was a significant difference between the infants’ pain in the intervention and the control groups. This difference can be explained by the fact that controlling sound and light as two physical stimuli have an effect on the changes in the Electroencephalogram of infants, and by reducing disturbing physical stimuli, pain responses in these infants can be reduced [11]. Also, unusual sensory stimuli such as light and noise in the environment can cause high excitability of the central nervous system of infants, especially premature infants who have an underdeveloped nervous system and increase their vulnerability to stimuli and lead to increased pain and Physiological inappropriate responses [17, 25]. Infants before birth in their mother’s womb are in an environment without light and minimal physical stimulation; however, after birth in the NICU environment, they are exposed to various fluctuations of light, sound, and other disturbing physical stimuli.

Wu et al. showed in their study that the pain caused by blood sampling in infants receiving the smell and taste of breast milk was less than in the group of infants under routine care [26]. Moreover, the pain in the other intervention group, which received the smell and taste of breast milk, and the mother’s heartbeat simultaneously, was less than the routine care group. The results of this study are similar to the results of our study because it was likewise shown in this study that using several simultaneous non-pharmacological interventions has a more palliative effect than one intervention alone.

In another study conducted by Alemdar and Ozdemir [13], the results showed that after venipuncture, the infants’ pain scores in the eye shield and intrauterine sounds groups were significantly different from the control group. Covering the premature infants’ eyes during blood sampling had a positive effect on the pain score after blood sampling. They recommended playing uterine sounds and covering premature infants’ eyes as easy, safe, and supportive methods during painful procedures. In addition, another study showed that the mean pain score of infants during and after placement of the orogastric tube in the intervention group (ear muffs and an eye shield) was significantly different from the control group [27], which is similar to the results of the present study. Aita et al. in a randomized trial entitled “The Effect of eye shield and earmuffs in reducing pain in premature infants”, showed that in infants who used earmuffs and eye shield before blood sampling, the pain intensity during blood sampling was not significantly different compared to the infants without eye shield and ear muffs, which is not consistent with the results of our study [17]. They attributed it to the existence of confounding factors, such as handling before the painful procedures and blood sampling.

Similarly, Mater et al. investigated the effect of eye shield compared to massage on premature infants’ response to pain during venipuncture. The results showed a significant difference between the pain intensity in the eye shield group, the massage group, and the control group during and after venipuncture. However, massage significantly reduced infants’ pain compared to eye shield [16]. In general, according to the findings of the present study and other studies, it can be concluded that the pain caused by painful procedures in infants can be reduced by using the combined non-pharmacological interventions of eye shield and ear muffs before blood sampling. The results of this study can be interpreted as follows: since the palliative effects of the combined interventions of eye shield and ear muffs are greater than using them alone and considering that using eye shield and ear muffs is a straightforward, comfortable and inexpensive method, therefore, to provide more comfort for infants during painful procedures, it is recommended to use these two interventions simultaneously.

Our study showed that heart rate changes were significant among the studied groups during and after blood sampling. In other words, there was a significant difference between the heart rate of infants during blood sampling in the eye shield and the control groups, the ear muffs and the control groups, and the eye shield plus ear muffs group and the eye shield, ear muffs, and the control groups. Abujarir et al.‘s study in Qatar showed that using ear muffs in the NICU had a positive effect on the infants’ vital signs. That is, during routine activities in the NICU, the heart rates in the group of infants with ear muffs were lower than that of infants without ear muffs [28]. Mater et al.‘s study showed that using eye shield compared to massage in premature infants during venous blood sampling decreased heart rate [16]. While the study by Alemdar and Ozdemir showed that after venipuncture, the heart rate of infants in the eye shield and intrauterine sounds groups was not significantly different from the control group [13]. Moreover, in Aita et al.‘s study, heart rate changes were not significant in the intervention (ear muffs and eye shield) and control groups during blood sampling, which is inconsistent with our results. The reason may be due to the type of ear muffs, eye shield, or confounding factors [17].

According to the findings of this study, the percentage of blood oxygen saturation of infants during the intervention in the ear muffs and the ear muffs plus eye shield was significantly different from the control group. Furthermore, the percentage of saturated oxygen after the intervention in the eye shield, ear muffs, and eye shield plus ear muffs groups significantly differed from that of the control group. Abujarir et al.‘s study showed that using ear muffs in NICU infants was associated with a significant increase in oxygen saturation level [28]. Besides, the study by Pourarin et al. showed that exposure to the smell of breast milk could effectively reduce the need for oxygen therapy by affecting the percentage of oxygen saturation, which is consistent with the present study [29]. While the study by Mater et al. showed that, compared to massage, using eye shield in premature infants during venous blood sampling had no effect on the percentage of oxygen saturation [16].

One of the strengths of the present study is its randomized clinical trial design with a control group and the approach of applying non-pharmacological pain management in infants, which is rarely conducted. One of the limitations of this study is the presence of extra ward noise during the morning shift due to the alarms of devices and equipment and the crowding of personnel and students in the ward, which may affect newborns’ physiological indicators. Another limitation of the study was the slight difference in the pain score before the intervention in the groups. Although this difference was not statistically significant, it is suggested to conduct studies with a larger sample size in the future.

## Conclusion

The present study provides clinical evidence that the concurrent use of ear muffs and eye shield are more effective than each intervention alone in controlling the infants’ pain during painful procedures. Since the adjustment of environmental stimuli, such as light and sound, is one of the methods of pain control and the use concurrent of ear muffs and eye shield is one of the convenient, effective, practical, and inexpensive methods. Therefore, it is recommended when performing venous blood sampling in the NICU, the concurrent use of ear muffs and eye shield should be used for better pain relief.

## Data Availability

The datasets generated and/or analyzed during the current study are not publicly available due [individual privacy could be compromised] but are available from the corresponding author on reasonable request.

## Abbreviations

**HR**: Heart Rate
**HRV**: Heart rate variability
**NIPS**: Neonatal Infant Pain Scale
**NICU**: Neonatal Intensive Care Unit
**VLBW**: very low birth weight
